# Mechanical Ventilation Injury and Repair in Extremely and Very Preterm Lungs

**DOI:** 10.1371/journal.pone.0063905

**Published:** 2013-05-21

**Authors:** Nadine Brew, Stuart B. Hooper, Valerie Zahra, Megan Wallace, Richard Harding

**Affiliations:** 1 Department of Anatomy and Developmental Biology, Monash University, Clayton, Victoria, Australia; 2 The Ritchie Centre, Monash Institute of Medical Research, Monash University, Clayton, Victoria, Australia; 3 Department of Obstetrics and Gynaecology, Monash University, Clayton, Victoria, Australia; University of Giessen Lung Center, Germany

## Abstract

**Background:**

Extremely preterm infants often receive mechanical ventilation (MV), which can contribute to bronchopulmonary dysplasia (BPD). However, the effects of MV alone on the extremely preterm lung and the lung’s capacity for repair are poorly understood.

**Aim:**

To characterise lung injury induced by MV alone, and mechanisms of injury and repair, in extremely preterm lungs and to compare them with very preterm lungs.

**Methods:**

Extremely preterm lambs (0.75 of term) were transiently exposed by hysterotomy and underwent 2 h of injurious MV. Lungs were collected 24 h and at 15 d after MV. Immunohistochemistry and morphometry were used to characterise injury and repair processes. qRT-PCR was performed on extremely and very preterm (0.85 of term) lungs 24 h after MV to assess molecular injury and repair responses.

**Results:**

24 h after MV at 0.75 of term, lung parenchyma and bronchioles were severely injured; tissue space and myofibroblast density were increased, collagen and elastin fibres were deformed and secondary crest density was reduced. Bronchioles contained debris and their epithelium was injured and thickened. 24 h after MV at 0.75 and 0.85 of term, mRNA expression of potential mediators of lung repair were significantly increased. By 15 days after MV, most lung injury had resolved without treatment.

**Conclusions:**

Extremely immature lungs, particularly bronchioles, are severely injured by 2 h of MV. In the absence of continued ventilation these injured lungs are capable of repair. At 24 h after MV, genes associated with injurious MV are unaltered, while potential repair genes are activated in both extremely and very preterm lungs.

## Introduction

Preterm infants often require mechanical ventilation (MV) for survival, especially those born at early gestational ages. For example, 62% of extremely preterm infants (defined as being born before 28 weeks’ gestation) in the USA received MV [1]. However, MV is a major contributing factor to bronchopulmonary dysplasia (BPD) [2], a chronic, inflammatory lung disease of preterm infants which can lead to long-term deficits in the respiratory health of survivors [3]. Although, the incidence of BPD varies widely between treatment centres, it is clear that the incidence increases with earlier gestational age at birth. In a multi-centre study of more than 18,000 very low birthweight infants born in the USA, 6–14% born weighing 1001–1500 grams (equivalent to appropriately grown 29–32 weeks gestation i.e. very preterm) developed BPD, while smaller infants, born weighing 501–1000 grams (equivalent to appropriately grown 25–28 weeks gestation i.e. extremely preterm), had a much greater risk of BPD, ranging from 33–46% [4]. As the requirement for MV [5] and the incidence of BPD [6] are both inversely related to gestational age, many more preterm infants with less mature (i.e. saccular stage) lungs require MV and subsequently develop BPD. Due to the greater incidence of MV and BPD in extremely preterm infants, compared to very preterm infants, it is necessary to understand the effects of MV alone on the lung at the saccular stage of lung development.

The effects of MV alone on the very immature lung have been difficult to determine because multiple interventions are required to maintain the life of preterm infants or experimental animals. While clinical studies of BPD provide vital information regarding disease manifestation and treatment, they are unable to delineate the precise role of MV, or any other single factor. In order to investigate the injurious effects of MV *per se* in the immature lung, and the underlying mechanisms, we developed a technique using fetal sheep which avoids potentially confounding factors, such as supplemental oxygen, glucocorticoids, impaired nutrition and surfactant. By ventilating the lungs of the ovine fetus with an intact placenta, we recently showed that a short period of injurious MV alone of the very immature, early-alveolar stage lung (0.85 of term) causes significant injury to the bronchioles and the future gas-exchanging region. Additionally, we reported that within 15 days and without any treatment, lungs were capable of virtually total repair after exposure to injurious MV [7].

The primary objective of our present study was to characterise lung injury manifestation and to investigate mechanisms of injury and repair following brief MV of the extremely preterm lung (i.e. saccular stage, 0.75 of term). A secondary objective was to compare the findings at 0.75 of term with our previous observations made in the early-alveolar stage lung at 0.85 of term [7]. The information we have obtained provides insights into the vulnerability of preterm infants at different stages of development to ventilator-induced lung injury (VILI) and BPD, and novel mechanistic insights into the ability of immature lungs to undergo self-repair. Tissue injury and repair were assessed in the lung parenchyma by documenting cell proliferation, myofibroblast differentiation and ECM deposition and in the bronchioles by assessing the epithelium and the presence of luminal debris 24 hours (h) and 15 days (d) after MV. In order to elucidate injury and repair processes at the molecular level, we assessed several molecular indicators of inflammation (IL-1β, IL-6, IL-8, TNF-α), VILI (CTGF, CYR61, EGR1) and tissue repair (MT2a, uPAR, DLK-1, HSPE-1).

## Materials and Methods

### Ethics Statement

The experimental protocol was performed in accordance with guidelines established by the National Health and Medical Research Council of Australia and was approved by the relevant Monash University animal ethics committee.

### Fetal Preparation and Treatment Groups

Under general anaesthesia (1.5% halothane in NO_2_–O_2_, 70∶30), aseptic surgery was performed on pregnant ewes at 110 d after mating (0.75 term; term is 147 d), as previously described [7]. After the head and chest of the fetus were exposed, a cuffed (3 mm diameter) endotracheal tube was inserted into the trachea via the mouth. Polyvinyl catheters were implanted into the carotid artery, jugular vein and amniotic sac. Fetal arterial blood gases and electrolytes were measured during MV and after surgical recovery to monitor fetal well-being.

### Treatment Groups

#### 1. Extremely preterm (saccular stage) short-term (24 h) group

This group was used to determine the early effects of MV. In this group, the ewe and fetus were euthanised (pentobarbitone sodium, 130 mg/kg i.v.) for lung tissue collection 24 h after MV (MV110+24 h, n = 6). Control fetuses for this treatment group underwent surgery but did not receive MV (C110+24 h, n = 7).

#### 2. Extremely preterm (saccular stage) long-term (15 d) group

This group was used to determine the prolonged effects of MV and the capacity for the lung to undergo repair. In this group, the ewe and fetus were euthanised for lung tissue collection 15 d after MV (MV110+15 d, n = 6), at 126 d gestation. Controls for this group (C110+15 d, n = 7) also underwent surgery and underwent necropsy at 126 d gestation.

#### 3. Very preterm (early-alveolar stage), short-term group

Lung tissue from fetal sheep at the early-alveolar stage of development was obtained from our previous study [7] and used for qRT-PCR analysis. MV injury and repair have been described in this cohort [7]. MV was performed at 125 d after mating (0.85 term) and lungs were collected after 24 h (MV125+24 h, n = 6); we used 8 age-matched controls (C125+24 h). Identical MV and tissue collection methods as described for animals in the present study were used.

### Mechanical Ventilation

MV, surgery and animal monitoring were performed as previously described [7]. The MV strategy was specifically chosen to induce lung injury. Briefly, fetal lung liquid was drained from the endotracheal tube and stored aseptically. Fetuses were mechanically ventilated (Dräger Babylog 8000+) for 2 h in “volume guarantee” mode targeting 5 ml/kg, using unhumidified air (0.21 FiO_2_, balance N_2_, ∼22°C), a peak inflation pressure of 40 cm H_2_O and an end-expiratory pressure of 0 cm H_2_O, and a frequency of 50 min^−1^. Throughout this time, the fetus was oxygenated naturally via the umbilical-placental circulation; fetal blood gas measurements confirmed adequate placental gas exchange. After 2 h of MV the fetus was returned to the uterus and fetal lung liquid replaced; the uterine incision was sutured closed and catheters were exteriorized through the ewe's flank.

### Lung Tissue Collection at Necropsy

At necropsy the fetal lungs were removed and weighed. The left bronchus was ligated, and portions of the left lung were snap-frozen at −70°C. The right lung was fixed for histology at 20 cm H_2_O with 4% paraformaldehyde infused via the trachea. Lung volume was determined as previously described [7]. Sections of right lung tissue were randomly selected for morphometric and immunohistochemical analyses and both injured and non-injured regions were included [7].

### Histological Staining

Sections of lung tissue were stained with hematoxylin and eosin for assessment of general lung morphometry, Hart's rescorcin-fuscin stain to identify elastin (14, 28) and Gordon and Sweet’s reticular stain to identify collagen.

### Immunohistochemical Staining

Proliferating cells and myofibroblasts were identified using Ki-67 (1∶100, M7240; DakoCytoMation) and α-smooth muscle actin antibodies (α-SMA, 1∶500, M0851; DakoCytoMation), respectively. Sections were incubated for 1 h using an immunohistochemistry kit (EnVision+ Dual Link System-HRP (DAB+) Dako Cytomation) according to manufacturer’s instructions, and sections were counterstained with hematoxylin to identify nuclei.

### Tissue Analysis

Methods for measuring tissue and airspace fractions, secondary septal crest density, and the staining density of elastin, collagen and α-SMA (a marker of myofibroblasts) have been previously described [7]. Three sections from different regions of the lung and five fields of view per section (15 in total) were analysed from each animal using image analysis software (ImagePro Plus). The number of Ki-67-labeled cells, expressed as the proportion of total cells, was used to determine the proportion of lung cells undergoing proliferation.

### Assessment of Bronchiolar wall Injury and Morphometric Analysis of Intact Bronchioles

The basement membrane perimeter of bronchioles (P_BM_) was used as an index of their size. The epithelial area of bronchioles and scoring of injury and luminal debris were quantified as previously described [8]. There was no difference in mean values of P_BM_ between treatment groups. For each parameter, a total of 15 randomly chosen bronchioles from three sections obtained from different regions of the lung, were analysed for each animal. All analyses were performed on coded slides by a single observer (NB) blinded to the experimental groups.

### Quantitative Real-time Polymerase Chain Reaction (qRT-PCR)

To provide information on lung injury processes at 24 h we measured mRNA levels of inflammation and early response genes that are highly expressed following MV. Expression of these genes was measured because they have been suggested to reflect the severity of lung injury as well as contributing to the development of lung injury [9], [10]. We measured tissue mRNA levels of connective tissue growth factor (*CTGF*), early growth response 1 (*EGR-1*), cysteine rich 61 (*CYR-61*), interleukins-1β (*IL-1*β), -6 (*IL-6*), and -8 (*IL-8*) and tumor necrosis factor-α (*TNF*-α), using quantitative real-time polymerase chain reaction (qRT-PCR) with ovine-specific primers [10]. To provide information about pulmonary tissue repair we measured mRNA levels of a subset of genes that have been shown to be significantly expressed in other studies of pulmonary repair [11]–[14]. Using qRT-PCR we measured mRNA levels of metallothionein (*MT2a*), urokinase plasminogen activator receptor (*uPAR*), delta-like homolog 1 drosophila (*DLK1*) and heat shock 10 kDa protein (*HSPE1*). Levels were expressed relative to expression of the ‘housekeeping’ gene 18 S to account for minor differences in sample concentration between animals. Total RNA was extracted, DNase-treated (RNeasy Maxi Kit; Qiagen) and one µg of RNA was reverse-transcribed into cDNA (Superscript III cDNA synthesis kit; Invitrogen). qRT-PCR was performed (Applied Biosystems 7900 HT real-time PCR machine) using reactions that contained cDNA template, forward and reverse primers, SYBR green (Power SYBR Green, Applied Biosystems) and nuclease-free water. qRT-PCR was used to measure gene expression as previously described [10] under optimised primer specific conditions (Table 1). The mRNA levels for each fetus were normalised to the 18 S rRNA values for that fetus and are expressed relative to the mean mRNA levels for that gene in the control group.

**Table 1:** Oligonucleotide primer sequences.

Gene	Genbank Accession #	Primer sequence
*CTGF*	DQ239672	F: 5′-TATAGCTCCAGCGACAGCTC-3′ R: 5′-ACGAACTTGACTCAGCCTCA-3′
*CYR-61*	DQ239628	F:5′-ATCGTCCAAACAACTTCGTG-3′ R: 5′-GGTAACGCGTGTGGAGATAC-3′
*EGR-1*	DQ239634	F: 5′-AGGGTCACTGTGGAAGGTC-3′ R: 5′-GCAGCTGAAGTCAAAGGAA-3′
*HSPE1*	BC102684	F: 5′-GCTCTAAAGGAAAGGGTGGA-3′ R: 5′-CTTTGGTGCCTCCATATTCTG-3′
*uPAR*	NM_001163606	F: 5′-TGCTGCTACTGCTGTTGGTT-3′ R: 5′-TCGTTGCGTTCTTACACTGG-3′
*MT2a*	NM_001075140	F: 5′-GGATCCCAACTGCTCCTG-3′ R: 5′-GCGCACTTGCAATCTTTG-3′
*DLK-1*	NM_174037	F: 5′-GGCATCGTCTTCCTCAACA-3′ R: 5′-GCAGCAGCAGGTTCTTCTT-3′
18S	X01117	F: 5′-GTCTGTGATGCCCTTAGATGTC-3′ R: 5′-AAGCTTATGACCCGCACTTAC-3′

CTGF, Connective tissue growth factor; CYR-61, cysteine rich 61; EGR1, early growth response 1; HSPE1, heat shock 10 kDa protein; uPAR, urokinase plasminogen activator receptor; MT2a, metallothionein 2a; DLK-1, delta like homolog drosophila; F, forward primer; R, reverse primer.

### Statistical Analysis

Numeric data are expressed as mean ± SE. For all morphometric analyses, comparisons were made using a nested ANOVA, with field of view, lung lobe, and treatment as factors. Data from qRT-PCR analyses were compared by t*-*test. Differences with *p* values <0.05 were considered statistically significant.

## Results

### Fetal Blood Gas and Electrolyte Status

At 2 h after the period of MV fetal arterial pH decreased (to 7.25±0.02) and lactate concentration increased (to 5.0±0.6 mmol/L at 2 h), likely due to maternal anesthesia and supine positioning. At 24 h after MV the fetal blood gas and electrolyte parameters were normal and stable (arterial pH 7.37±0.01 and lactate 1.7±0.2 mmol/L) and fetuses in the long-term survival study remained healthy until necropsy.

### Necropsy Data

Fetal body and organ weights, including wet and dry lung weights, were not different between MV and control groups both at 24 h and 15 d after MV (Table 2).

**Table 2:** Body weight, lung weights, DNA and protein concentration in saccular stage fetuses 24 h and 15 d after ventilation.

	Short Term Effects		Long Term Effects	
	C110+24 h	MV110+24 h	C110+15 d	MV110+15 d
**Body Weight (BW, kg)**	1.6±0.1	1.8±0.1	2.9±0.2	3.0±0.3
**Wet Lung Weight (g)/BW (kg)**	48±5	43±1	35±2	40±2
**Dry Lung Weight (g)/BW (kg)**	5.4±0.7	5.5±0.3	3.9±0.3	3.8±0.5
**Left Lung Volume (cm^3^)/BW (kg)**	59±3	51±3	45±4	46±4
**DNA Concentration (mg/kg BW)**	4.6±0.3	5.1±0.2	3.8±0.2	4.5±0.3
**Protein Concentration (mg/kg BW)**	38.2±3.9	38.0±3.3	30.9±4.6	27.8±2.3

Values are ± SE.

### DNA and Protein Concentration

The DNA and protein concentrations of lung tissue were not different between MV and control fetuses, both at 24 h and 15 d after MV (Table 2).

### General Lung Morphometry

**Figure 1:**
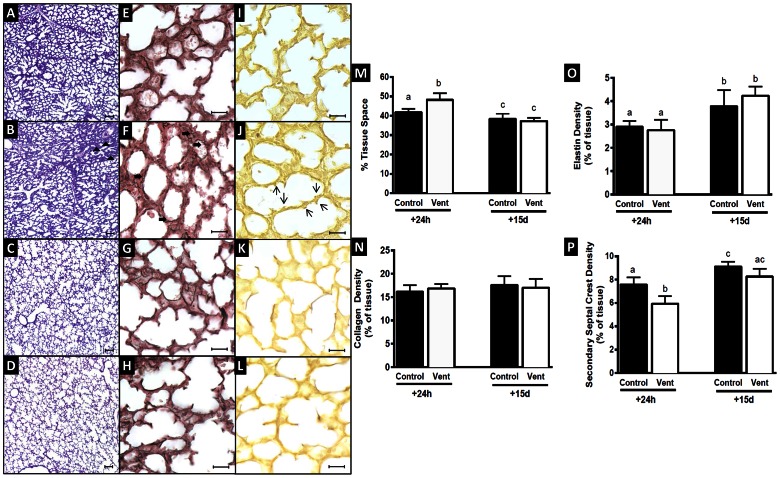
Lung morphometry, collagen and elastin density, percent tissue space and secondary septal crest density in saccular stage MV and control lungs after 24 h and 15 d. Light micrographs stained with hemotoxylin and eosin depicting lung morphology in C110+24 h (A), MV110+24 h (B), C110+15 d (C) and MV110+15 d (D) lung tissue. At 24 h after MV lung tissue showed signs of heterogeneous injury with regional hypercellularity and atelectasis (arrow, B). Tissue space fraction was increased in MV110+24 h lungs compared to controls (M, p<0.05). Collagen fibres (black staining) are shown in C110 d+24 h (E), MV110+24 h (F), C110+15 d (G) and MV110+15 d (H) and elastin deposits (brown staining) in C110+24 h (I), MV110+24 h (J), C110+15 d (K) and MV110+15 d (L). Collagen fibres (brown staining) were not straight in MV110+24 h lungs (arrow, F), compared to controls at both ages and MV110+15 lungs (E,G,H). Collagen (N) and elastin density (O) was not different between MV lungs and their matched control group. Secondary septal crest density was reduced in MV110+24 h lungs (arrow, J) compared to those in C110+24 h group (I, P). Scale bar = 100 µm for A–D and 20 µm for E–L. Values that do not share a common letter are significantly different.

At 24 h after MV, lung tissue showed heterogeneous injury. Injured regions of MV lungs displayed hypercellularity, regions of atelectasis (Fig. 1B) and regions containing erythrocytes, indicative of localised haemorrhage. Non-injured regions of MV lungs appeared structurally normal and similar to age-matched control lungs (Fig. 1A). In fetuses examined 15 d after MV, lung morphometry was not different from that of controls (Fig. 1C), with no detectable regions of injury (Fig. 1D).

### Percent Tissue Space

At 24 h the tissue fraction in the lungs of ventilated fetuses (48.3±3.5%) was significantly greater, by 17%, than in control fetuses (41.2±1.8%). By 15 d, there was no longer a difference in tissue fraction between MV (37.3±1.6%) and control lungs (38.3±2.7%). In both control and MV lungs the tissue fraction significantly decreased between 24 h and 15 d (Fig. 1M).

### Collagen Deposition and Abundance

Lungs of control fetuses at 24 h and 15 d contained thick, unfolded collagen fibres in the saccule walls and at the tips of developing septa (Fig. 1E). At 24 h after MV lung collagen fibres were highly folded and distributed throughout the distal parenchyma rather than being restricted to the saccular wall (solid arrows, Fig. 1F). By 15 d, collagen fibres in MV lungs were arranged as in control lungs (Fig. 1G,H). At 24 h, the relative abundance of collagen in lung tissue of MV fetuses (16.8±0.9%) was not different to that in the controls (16.1±1.5%). When quantified at 15 d, the relative abundance of collagen in MV fetuses (17.0±1.9%) was also not different to that of controls (17.5±1.9%). Between 24 h and 15 d, the relative abundance of collagen was not altered in control and MV fetuses (Fig. 1N).

### Elastin Deposition and Relative Abundance

At 24 h, elastin fibres in control lungs were deposited predominantly at the tips of developing septal crests (Fig. 1I). In MV lungs elastin was deposited in the tips of shorter, thicker septa and occasionally in the saccule wall (Fig. 1J). The relative abundance of elastin in lung tissue at 24 h in MV fetuses (2.8±0.4%) was not different to that of controls (2.9±0.2%). At 15 d, elastin was also present in saccular walls as well as at the tips of secondary septa. The relative elastin abundance in MV fetuses (4.2±0.4%) at 15 d remained the same as in controls (3.8±0.7%). Between 24 h and 15 d, relative elastin abundance in lung tissue increased significantly in both MV and control fetuses (Fig. 1O).

### Secondary Septal Crest Density

Secondary septal crests were recognised by elastin deposits at their tips. At 24 h secondary crests appeared stunted and thicker in MV fetuses than in controls, and occupied a significantly smaller proportion of lung tissue (5.9±0.6%, open arrows, Fig. 1I) than in controls (7.6±0.6%). Fifteen days later secondary crest density and morphology was not different between MV (8.3±0.7) and control lungs (9.1±0.4%). Between 24 h and 15 d, secondary crest density in control fetuses underwent a significant increase (∼20%). MV fetuses (with diminished secondary crest density at 24 h) underwent a larger increase (41%) in secondary septal crest density over the same period (Fig. 1P).

### Cell Proliferation

**Figure 2:**
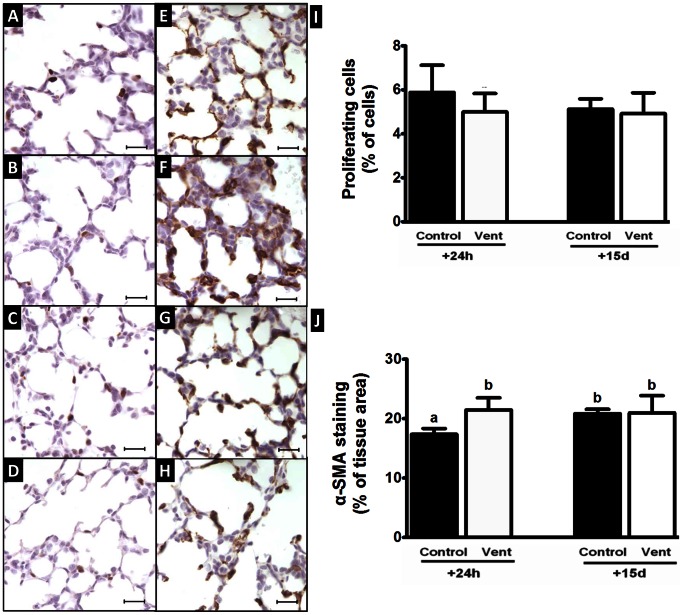
Cell proliferation and myofibroblast density in saccular stage MV and control lungs after 24 h and 15 d. Immunohistochemical staining using Ki-67 antibody shows proliferating cells labelled brown in C110+24 h (A), MV110+24 h (B), C110+15 d (C) and MV110+15 d lungs (D). The proportion of proliferating cells in the gas-exchanging region was not different between MV lungs and there matched control groups (I). Myofibroblasts were detected using α-SMA antibody, which labelled the myofibroblasts brown as shown in C110+24 h (E), MV110+24 h (F), C110+15 d (G) and MV110+15 d lungs (H). Myofibroblasts were localised at developing septa. α-SMA was increased in MV110+24 h lungs, in comparison to controls (J, p<0.05). Scale bar = 20 µm. Values that do not share a common letter are significantly different.

The cellular proliferation rate in lung parenchyma of MV fetuses at 24 h (5.0±0.8%) was similar to that of controls (5.9±1.2%). At 15 d the cellular proliferation rate was also similar between the MV (4.9±1.0%) and control fetuses (5.1±0.5%). The cellular proliferation rate in lung parenchyma did not change between 24 h and 15 d in either the control or MV groups (Fig. 2I).

### Relative Abundance of Myofibroblasts in Lung Parenchyma

In the distal lung, myofibroblasts, identified by α-SMA staining were present at the tips of developing septa and saccule wall in all groups. At 24 h the relative abundance of α-SMA staining in MV lungs was significantly greater (21.5±2.0%) than in control lungs (17.4±1.0%). Fifteen days later the relative abundance of α-SMA staining was not different between MV (21.0±2.9%) and control fetuses (20.9±0.7%, Fig. 2J).

### Bronchiolar Analysis

**Figure 3:**
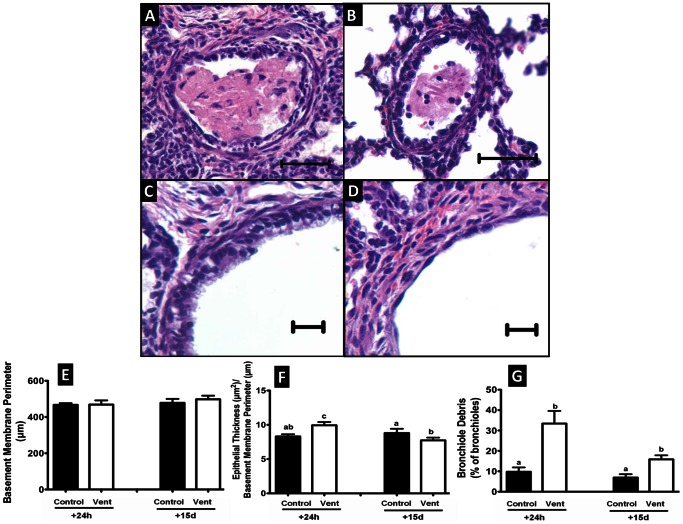
Morphological and injury analysis of bronchioles in saccular stage MV and control lungs after 24 h and 15 d. Light micrographs show cellular intraluminal debris in MV110+24 h lungs (A, B), intact epithelium of C110+24 h bronchiole (C) and bronchiole with denuded epithelium in MV110+24 h lung (D). The basement membrane perimeter of bronchioles was not different between all saccular stage MV and control lungs (E). Epithelial thickness of bronchioles was greater in MV110+24 h fetuses than in controls; however, it was lower in MV110+15 days fetuses relative to 15 days controls (F). The proportion of bronchioles that contained debris within the lumen was increased in MV110+24 h and MV110+15 d fetuses compared with age-matched controls (G). Values that do not share a common letter are significantly different from each other (P<0.05, scale bar = 10 µm in A and B and 20 µm in C and D).

The mean perimeter of the basement membrane (P_BM_) of bronchioles analysed was not different between control fetuses at 24 h (467±9 µm) or 15 d (478±22 µm), and these values were not different to those of MV fetuses at 24 h (469±23 µm) or 15 d (498±20 µm, Fig. 3E).

At 24 h, the area of bronchiolar epithelium relative to P_BM_ in fetuses exposed to MV (9.9±0.5 µm^2^/µm) was significantly greater than in controls (8.3±0.3 µm^2^/µm). At 15 d the epithelial area of MV bronchioles (7.7±0.4 µm^2^/µm) was significantly smaller than in controls (8.8±0.6 µm^2^/µm). In control fetuses the bronchiolar epithelial area relative to PBM did not change between 24 h and 15 d, but it decreased in MV fetuses (Fig. 3F). At 24 h the lumen of 33.4±6.2% of MV bronchioles contained cellular and acellular debris; the debris appeared to consist of inflammatory cells, proteinaceous exudate and detached airway epithelium (Fig. 3A, B, G). At 24 h, there was less luminal debris in control fetuses (9.8±2.1%, p<0.05) than in MV fetuses (33.4±0.6%) and it accounted for a smaller proportion of luminal space. At 15 d the proportion of bronchioles containing debris in MV lungs (15.9±2.0%) was lower than at 24 h, although still greater than in controls (6.9±1.7%).

In ventilated fetuses epithelial injury was present in 34.9±6.5% of bronchioles 24 h after MV compared to only 2.0±1.5% in the controls. Seventy-five per cent of injured bronchioles in MV fetuses were severely injured; i.e. >180° of the epithelium was affected (Table 3). At 15 d the proportion of bronchioles that were injured in MV lungs was only 4.3±2.3%, and no longer different to the proportion measured in controls (3.3±1.9%).

**Table 3:** Injury analysis of saccular stage control and ventilated bronchioles after 24 h and 15 d.

	Group	Fetuses(n)	AirwaysAnalysed (n)	Intact(n)	TotalInjured (n)	% Injured	Mild Injury(n/%)	ModerateInjury (n/%)	SevereInjury (n/%)
**Short Term** **Effects**	**C110+24** **h** **MV110+24** **h**	7 6	217 199	213 132	4 67*	2.0 34.9*	3 (75%) 7 (10%)	1 (25%) 10 (15%)	0 (0%) 50 (75%)*
**Long Term** **Effects**	**C110+15** **d** **MV110+15** **d**	6 7	216 180	199 172	7 8	3.3 4.3	4 (57%) 4 (50%	3 (43%) 2 (35%)	0 (0) 2 (25%)

Mild injury: 45° bronchiole epithelium detached or absent; moderate: 45°–180° bronchiole epithelium detached or absent; severe: 180° bronchiole epithelium detached or absent. Injured data represent total no. of mild, moderate, and severely injured bronchioles. MV110+24 h lungs had a higher proportion of injured bronchioles relative to all other groups, of which most were classified as severely injured (p<0.05).

### mRNA Levels of “repair” Genes

**Figure 4:**
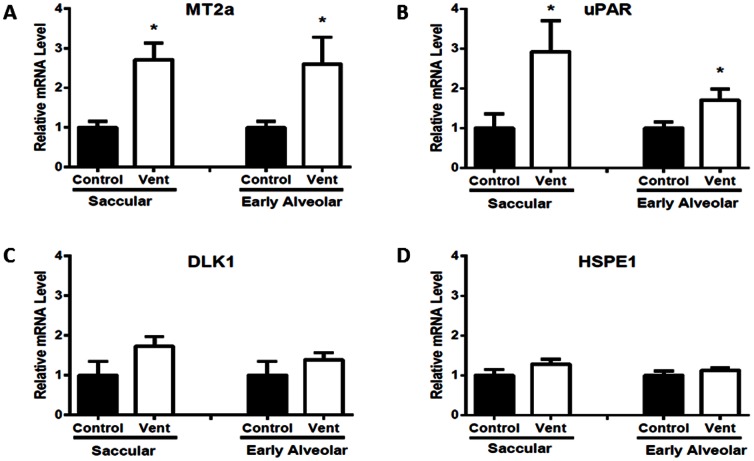
Relative expression of potential repair gene mRNA in saccular and early alveolar stage lungs 24 h after MV. Metallothionein (A) and Urokinase Plasminogen Activator Receptor (B) mRNA expression was significantly increased in MV saccular and early alveolar stage lungs after 24 h when compared to controls (p<0.05). Relative expression of Delta-Like Homolog Drosophila (C) and Heat Shock 10 kDa Protein (D) mRNA was not different between control and MV lungs at 24 h in the saccular or early-alveolar stage lung.

MV significantly increased mRNA levels of MT2a and uPAR 24 h after MV in both saccular (MV110+24 h) and early alveolar stage (MV125+24 h) lungs (1.7–2.9 fold increases in mRNA expression) when compared to respective controls. HSPE1 and DLK1 mRNA levels were not different 24 h after MV in saccular (MV110+24 h) or early-alveolar stage (MV125+24 h) lungs when compared to respective controls (Fig. 4).

### mRNA Levels of Inflammatory and Early Response Genes

mRNA levels of inflammatory genes (IL-1β, IL-6, IL-8 and TNF-α) and early response genes associated with lung injury (CTGF, CYR61, EGR1) in saccular stage lungs were not different 24 h after MV compared to respective controls (Table 4). The mRNA levels of these genes in very preterm early-alveolar stage lungs at 24 h have been previously published [7] and were not different between control and MV fetuses.

**Table 4:** The mRNA levels of control and ventilated saccular lungs after 24 h, corrected for the level of housekeeping gene 18 S and expressed as fold change from the mean value in control fetuses ± SE.

	Pro-Inflammatory genes				VILI genes		
	*IL-1β*	*IL-6*	*IL-8*	*TNF-α*	*CTGF*	*CYR61*	*EGR1*
**C110+24** **h**	1.0±0.3	1.0±0.3	1.0±0.3	1.0±0.1	1.0±0.3	1.0±0.1	1.0±0.3
**MV110+24** **h**	0.6±0.3	0.5±0.1	0.6±0.1	1.3±0.3	1.6±0.3	0.9±0.2	1.6±0.6

IL, interleukin, TNF-α tumor necrosis factor-α; CTGF, connective tissue growth factor; CYR61, cysteine-rich 61; EGR1, early growth response 1.

## Discussion

### Effect of MV alone on the Extremely Preterm Lung

We have shown that only 2 hours of MV of the extremely preterm, saccular stage lung can cause considerable injury to bronchioles and the lung parenchyma. At 24 h after MV the injured lungs had a greater tissue fraction, decreased secondary septal crest density, increased myofibroblast density, altered collagen deposition in the saccular walls, and a reduced percentage of proliferating cells. A high proportion of MV bronchioles had injured epithelium, and in those that remained intact the epithelium was thickened. There was also a significant increase in bronchiolar luminal debris. However, at 15 d after MV the lung parenchyma and bronchioles had undergone substantial repair, with very little evidence of injury. The finding of spontaneous repair in the extremely preterm, saccular stage lung is in accordance with our previous report in the very preterm early-alveolar stage lung [7]. However, there were differences in the injury pattern induced by MV at the two developmental stages. In saccular stage lungs the bronchioles were most injured by MV, while in the more mature early-alveolar stage lungs it was the parenchyma region that was most affected. Our study strongly suggests that the developing lung has a remarkable ability to recover from brief severe MV-induced injury when left unperturbed.

### Differential Effects of MV Injury in Extremely versus Very Preterm Lung

We used an identical MV strategy to that used previously to induce lung injury in very preterm early-alveolar stage lungs. As the incidence of BPD increases inversely in relation to birth weight and gestational age [4] it may be expected that the distal lung parenchyma would be more injured at the earlier gestational age. However, we found that MV-induced injury in the distal parenchyma of extremely preterm, saccular stage lungs was less severe than in early-alveolar stage lungs [7]. Decreased secondary septal crest density and increased tissue space fraction was less pronounced in saccular stage lungs after MV, compared to early-alveolar lungs. However, in the bronchioles, luminal debris was three times more prevalent in saccular lungs exposed to MV when compared to controls, while in early-alveolar stage lungs exposed to MV there was only a 2-fold increase in the incidence of luminal debris. The degree of bronchiolar injury in the saccular stage lungs was of a similar severity to that seen in early-alveolar stage lungs [7].

The differences in MV-induced injury between saccular and early-alveolar stage lungs may be due to age-related differences in the compliance of the conducting airways and lung parenchyma, as the airways become less compliant and parenchyma more compliant with increasing gestational age [15]. If the airways are highly compliant, pressure and hence volume changes may dissipate at the level of the airways and therefore may be less severe in the distal airspaces. That is, the attenuated expansion and deflation of the terminal saccules with each breath may protect them from injury. Studies of airways of preterm infants with BPD [16] and preterm lambs receiving MV [17] have shown that injury of the conducting airways commences at the trachea and descends along the airway tree to more distal airway generations. Using a similar technique to that of the present study, brief, injurious MV (15 min) of preterm lambs at 129 days gestation caused injury in the large airways and bronchioles without causing extensive injury to the distal lung [17].

### Potential Mechanisms of Repair in the Extremely and Very Preterm Lung

Because immature lungs at both stages of development were capable of substantial structural repair in our VILI model after 15 d we were able to assess potential mediators of repair and injury activated at 24 h after MV. Our study has shown that within 24 h of injurious MV of both very and extremely preterm lungs, repair processes have already begun, including the normalisation of injury and pro-inflammatory gene expression and increased expression of genes involved in tissue repair. The expression of two genes previously associated with pulmonary repair in other models of lung repair, metallothionein (MT) and urokinase plasminogen activator receptor (uPAR) [11], [12], [14], were significantly increased at 24 h after MV at both stages of development; this suggests that these genes may contribute to lung repair in our model.

MT is a strong antioxidant [18], [19] induced by metals, glucocorticoids, oxidative stress and inflammatory mediators [18] and its expression has previously been reported in lungs of humans [20], lambs [21] and mice [22]. There is evidence that MT is involved in lung repair and the prevention of lung injury. Knockout mice lacking MT are more sensitive to acute lung injury caused by lipopolysaccharide [23], ozone [24] and allergic airway inflammation [25]. MT expression is greatest in bronchiolar epithelium, alveolar macrophages and endothelial cells [20], [21].

uPAR is part of the plasminogen activation system and its gene expression was elevated after MV. Under physiological conditions plasminogen and plasmin activation are critical for fibrinolysis and clot removal [26]. In infants with RDS, fibrinolytic activity is depressed in tracheal aspirates taken on their first day of life and even further depressed in infants with RDS who go on to develop BPD, when compared to control infants [27]. We speculate that increased expression of uPAR mRNA within MV lungs at 24 h may promote the synthesis of plasminogen and plasmin, mediated by uPAR, resulting in enhanced fibrinolysis, clearance of fibrin and hence lung tissue repair.

### VILI in Extremely Preterm Lung: Effects on Lung Architecture, Extracellular Matrix and Bronchioles

Collagen fibres were highly folded and thinner in MV lungs compared to controls. Disrupted collagen architecture has also been reported in infants with BPD [28], [29]. In adults, MV is associated with lung fibrosis [30]; however it is not clear whether BPD in infants is a fibrotic condition [29], [31], [32]. In the present study of the saccular stage lung, as well as in the early-alveolar stage lung [7], brief MV disrupted collagen architecture, although it did not affect the abundance of collagen measured in the distal lung. At 24 h after MV, elastin was predominantly deposited at the tips of short, thickened, secondary septal crests. BPD in infants [33] and animal models of BPD [32], [34], [35] are characterised by an increased abundance of pulmonary elastin in the distal lung. As in our previous study of early-alveolar stage lung, brief, injurious MV of the saccular stage lung did not stimulate an increase in the relative abundance of elastin in the distal lung [7]. Increased expression of tropoelastin mRNA and the deposition of elastic fibres appears dependent on MV duration [36]. It is possible that 2 h of MV is too brief a period to induce significantly increased elastin and collagen synthesis. Alternatively, collagen and elastin synthesis are likely to have increased in proportion to the tissue space increase we measured, resulting in no net increase in the relative abundance these ECM components.

Secondary septal crests are highly susceptible to MV-induced injury [34], [35], [37], [38]. The reduction in secondary crest density following MV was only approximately half as great in the saccular stage lung as in the early-alveolar stage lung (22% in saccular lung vs. 40% in early-alveolar stage lung). Secondary crests in the early-alveolar stage lung are likely to be both more abundant and more vulnerable to MV damage because they are thinner and longer than those in saccular stage lung. Secondary septal crest density in the saccular lung was restored to control levels by 15 d after MV, as is the case in early-alveolar stage lung [7].

Consistent with previous studies [8], [39], we found that MV caused severe epithelial damage in immature bronchioles. Thickening of the bronchiolar epithelium has also been reported in infants with BPD [40] and preterm baboons [41] as well as lambs [8] that received MV. Furthermore, children with BPD have persistently impaired airway function [3], [42], reduced peak expiratory flow [43] and an increased asthma risk [44], [45], all strongly suggesting that BPD pathogenesis severely affects the conducting airways in addition to alveoli.

### Role of Myofibroblasts in VILI in Extremely Preterm Lung

Myofibroblasts secrete ECM molecules such as collagen and elastin and are critical for lung repair and alveolarization, as well as contributing to pathogenic remodelling. MV has been shown to increase myofibroblast abundance in a preterm primate model of BPD [46]. Increased myofibroblast abundance has also been described in infants exposed to MV [47], [48] as well as in other ovine models of MV-induced injury [32], [34]. Myofibroblast abundance was increased in saccular stage lung 24 h after MV; this was also found by Allison et al at 12 h after brief MV (1 h and 6 h) of saccular stage ovine lung [34]. In the early-alveolar stage lung, however, the relative myofibroblast abundance was not different to control levels at 24 h [7], indicating that myofibroblast stimulation may be dependent on the stage of lung development.

### Molecular Mediators of VILI in Extremely Preterm Lung

Pro-inflammatory cytokines and the early response genes evaluated in this study have previously been strongly associated with the pathogenesis of lung injury [10], [49]–[52]. By 24 h after MV, mRNA expression levels of pro-inflammatory cytokines and early response genes were not different between MV and control fetuses, indicating the cessation of the acute phase of lung injury. Based on our previous experiments [10], [49] and those of other investigators [53], it appears probable that the expression of early response genes and pro-inflammatory cytokines was elevated during MV, and then normalised by 24 h. Even very brief injurious MV (e.g. 15 min) of preterm lamb lungs has been shown to dramatically increase CTGF, CYR-61 [10], [54], EGR-1, IL-1ß, IL-6, and IL-8 mRNA levels [10] in the distal lung. Decreased expression or normalisation of pro-inflammatory cytokines and early response genes following the initiation of MV has also been reported [10]. In preterm infants (<30 weeks GA) circulating plasma IL-6 and IL-8 levels fall by day 3 in the presence of continued MV [55], [56].

### Conclusions

MV of the extremely immature, saccular stage lung causes injury in the distal lung, which is most severe in bronchioles. Our study suggests that the bronchioles of extremely preterm infants may be at greater risk of MV-induced injury than those of infants with more developed lungs. Despite differences in lung injury manifestation between saccular and early-alveolar stage lung, our findings indicate that both the extremely preterm and very preterm lung have the capacity for repair after brief injurious MV, if left unperturbed. At 24 h after MV, although lungs are severely injured, it is apparent that repair processes have commenced, manifestation of acute-phase lung injury has ceased, and that the saccular and early-alveolar stage lungs are likely to undergo repair by similar mechanisms.
